# Dietary behavior of video game players and esports players in Germany: a cross-sectional study

**DOI:** 10.1186/s41043-023-00373-7

**Published:** 2023-04-06

**Authors:** Markus Soffner, Peter Bickmann, Chuck Tholl, Ingo Froböse

**Affiliations:** Institute of Movement Therapy and Movement-Oriented Prevention and Rehabilitation, German Sport University Cologne, Cologne, Germany

**Keywords:** Gaming, Food frequency questionnaire, Nutrition, Well-being

## Abstract

**Background:**

Video gaming and competitive gaming (esports) are gaining more and more recognition in society as well as in research. Increasingly, health-related topics are the focus of research on video game and esports players. Although video gaming is often associated with energy drinks and fast food, no studies have yet examined the players’ dietary behavior. Therefore, the aim of this cross-sectional study is to investigate the dietary behavior and additional health-related data of video game players and esports players in Germany.

**Methods:**

Between July and October 2020, 817 participants (87.1% male; 24.2 ± 6.9 years), divided into video game players and esports players, were surveyed via an online questionnaire about their dietary, health, and gaming behaviors. Descriptive statistics were performed on all questions. To investigate statistically significant differences between video game players and esports players, the Mann–Whitney-U-Test and Kruskall-Wallis-Test were used. Partial Spearman correlations were used to examine possible associations between dietary behavior, health status, well-being, and video game playing time.

**Results:**

Water was the primary source of fluid intake for the players (10.9 ± 7.0 l/week). The average weekly consumption of energy drinks was 0.4 ± 0.9 L. Energy drinks (rho = 0.14; p < 0.01) as well as soft drinks (rho = 0.14; p < 0.01) are positively correlated with the video game playing time. Participants ate 7.5 ± 10.4 servings of fast food per month, which has a positive association with video game playing time (rho = 0.13; p < 0.01). In contrast, vegetables (1.7 ± 1.6 servings/day) and fruits (0.9 ± 1.0 servings/day) are eaten almost daily.

**Conclusion:**

In this survey, the dietary behavior of video game players and esports players is similar to that of the German general population. Nevertheless, there is a need for improvement. Especially energy drinks, which are already documented to have adverse health effects, should be limited. In addition, the consumption of fast food and meat should also be reduced, and healthier foods such as fruits and vegetables should be increased instead. Early education and support regarding the associated risks with unhealthy foods is important within the target group.

## Introduction

Competitive gaming (esports) and video gaming have long ceased to be a simple trend. In recent years, the field has grown steadily, gained more and more recognition in society, and is becoming increasingly popular as a recreational activity. The numbers of active players and people who watch video games via streams are rising every year [1]. The COVID-19 pandemic gave a particular boost to gaming. Since the outbreak, the time spent gaming has significantly increased [2].

The rising interest in gaming is also reflected in research. Since 2015, publications have grown consistently [3]. Scientists are becoming increasingly concerned about people who play video games, particularly from a health standpoint, and are conducting extensive research into the health of gamers. The high playing times are a particular risk [4]. Because video gaming is a screen-based activity like working at a computer, this activity is characterized by long periods of sitting and is often accompanied by a sedentary lifestyle [5, 6]. This often results in musculoskeletal disorders [7]. Furthermore, long periods of sitting are already recognized as a risk factor for numerous chronic diseases [8–10] and all-cause mortality [11–13]. For these reasons, not only compensatory exercise but also a balanced and healthy diet can somewhat counteract these risks. However, the dietary behavior of gamers has not been studied so far. Clichés about gamers’ excessive consumption of fast food and energy drinks exist. Increased consumption of this can lead to weight gain and diseases like diabetes [14–16]. Overall, unhealthy nutrition can be the cause of obesity [17], heart diseases [18], or metabolic disorders [19, 20]. As a result, the target group of video game players and esports players is at greater risk when combined with a sedentary lifestyle.

However, there is a lack of evidence when examining the diet of gamers. Hence, the aim of this exploratory study is to investigate the dietary behavior of video game players and esports players in Germany. Additionally, health behaviors and body image were surveyed. Collecting data on the players’ dietary behavior is essential for providing specific health promotion to this at-risk target group over the long term.

## Materials and methods

### Study design and setting

The current study is a successor to the eSports Study 2019 [21] and 2020 [22] with a different focus and was carried out as a cross-sectional online survey. The data collection took place from July to October 2020. Due to the COVID-19 pandemic, the questionnaire was distributed exclusively online in digital form. For this purpose, video gaming and esports platforms, the project-related website (*esportwissen.de*), and social media (*Facebook, Instagram, Discord*) were used. In addition, esports organizations were contacted, and gaming websites wrote articles about the survey to further promote it. To motivate potential participants for the survey, the aim of the study and eligibility criteria were explained during the distribution. For an additional incentive to participate in the survey, participants were able to enter a voluntary raffle after completion (e.g., *one out of five 20-euro Amazon vouchers*). The study size was based on the previous studies and aimed to reach around 1000 participants during the survey period. The ethical committee of the German Sport University Cologne approved the study (reference: 053/2018).

### Participants

Participants were eligible if they lived in Germany at the time and understood the German language in order to reach only the German gaming scene. Other participation requirements included being 14 years old or older and actively playing video games. The first page of the survey showed an information sheet specifying the purpose of the study, and consent to participate was obtained. Before the actual survey started, the participants were asked about their country of residence and age. For participants who did not live in Germany or who were younger than 14 years, the survey ended, and they were excluded.

### Measures

The questionnaire was designed to examine the dietary behavior of video game players and esports players. Demography, video gaming behavior, and health behavior were also included as topics. Overall, the questionnaire contained a total of 61–63 questions, depending on the participants’ answers to the filter questions. It was created and conducted using the online survey tool Unipark (*Questback GmbH, Cologne, Germany*).

At the beginning, participants were asked about their demographic and anthropometric data. The wording and rating of these questions were designed in accordance with the standards of the German Federal Statistical Office [23].

Subsequently, gaming behavior was investigated with self-designed questions, because no appropriate and validated questionnaires were available. First, participants had to classify their player status:

- professional players (regularly earning significant revenue from esports, such as prize money, sponsorships, and salaries from clubs),
- former professionals (esports professionals who no longer compete),
- amateurs (playing esports, but without earning a significant amount of money),
- regular players (playing video games or esports more than once a week, but without taking part in official tournaments and leagues),
- occasional players (playing video games several times a month or less without participating in official tournaments and leagues),
- non-players.

Moreover, participants should indicate their favorite game genre (a semi-open-ended question) and their video game playing times (hours per week). The game time was divided into four categories:

- alone against human opponents (player vs. player),
- with human players together against human opponents (koop-player vs. player),
- alone against computer-controlled opponents (player vs. environment),
- with human players together against computer-controlled opponents (koop-player vs. environment).

The questions on the health behaviors of the participants referred to their last four weeks. The self-rated health was measured with the first question of the *SF-36* questionnaire [24]. The duration of moderate- to vigorous physical activity was reported in hours per week and sedentary time in hours per day. Furthermore, the entire version of the *WHO-Five Well-Being Index* [25] was implemented in order to assess individual well-being.

The main part was introduced with two questions referring to the diet of the participants:

- If they are vegan or vegetarian,
- Which foods they avoid (multiple possible answers: "meat, poultry, or sausage," "fish," "milk or dairy products," "eggs").

The dietary behavior was surveyed according to the *food frequency questionnaire* of the Robert-Koch Institute [26]. It can be separated into two parts: the dietary behavior and the drinking behavior. The questionnaire was slightly modified. Certain foods were combined (e.g., whole grain bread, mixed bread, and white bread as bread in general) or excluded (e.g. consumption of jam or honey) to make the questionnaire more time efficient.

**Fig. 1:**
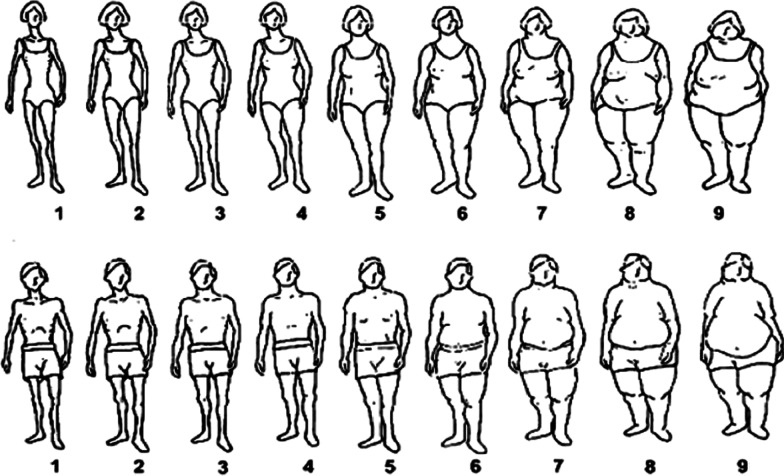
Body image scale [27]

The last part of the questionnaire consisted of the participants’ personal body image and their desired body image. For this purpose, the body image scale of Stunkard, Sørensen, and Schulsinger (1983) was used (Fig. 1).

### Data analyses

The data were checked for completeness, consistency, and plausibility. Participants were excluded from all analyses if their data was inconsistent (e.g., reporting to be a high school student with a bachelor’s degree) or implausible (e.g., incredible high amounts of foods and drinks consumption). Participants were also excluded if they did not complete the full survey. In the case of missing data for individual questions, the participant was excluded only for the corresponding parameter. For calculations, list wise exclusion was chosen to maintain a consistent number of subjects in the tables.

For a clearer analysis of the dietary data and more clearly arranged tables, the player groups were grouped into video game players and esports players. The esports players include professionals and amateurs. All other groups were summarized in the video game group. The term “gamers” is used for both groups together. Furthermore, for clarity, the foods related to dietary behavior were grouped together in the tables.

The participants' weight and height were measured, and their body mass index (BMI) was calculated. The playing times of the individual game categories were added together to obtain the total playing time. The amount of drinking and food consumption was recorded monthly, which corresponds to 28 days. For daily consumption, the servings were calculated down. For the calculations of the correlations with body image, first the difference between the desired body image and the current body image was calculated. Based on this, the differences were categorized from 1 = agreement to 9 = greatest difference.

Descriptive statistics were applied to all the data. For each player status, numerical data as well as ordinal data were described by means, standard deviations, median and IQR. Nominal data were described by frequencies. All data were checked for normal distribution. If a non-normal distribution was present, statistical differences between the groups were calculated by the Kruskal–Wallis test, Fisher’s exact test, or Mann–Whitney U-test. Otherwise, ANOVA was performed. Post-hoc tests with Bonferroni correction were performed if overall group differences were statistically significant. Sign test was used to calculate differences between the current body image and the desired body image. To examine possible associations, partial Spearman correlations were calculated. The resulting coefficients were interpreted according to Cohen [28] as poor (rho = 0.10–0.29), moderate (rho = 0.30–0.49), and strong (rho > 0.50). The level of significance for all analyses was set at p < 0.05. All statistical analyses were conducted using IBM SPSS Statistics 26 (IBM Corp., Armonk, NY, USA).

## Results

### Participants

**Fig. 2:**
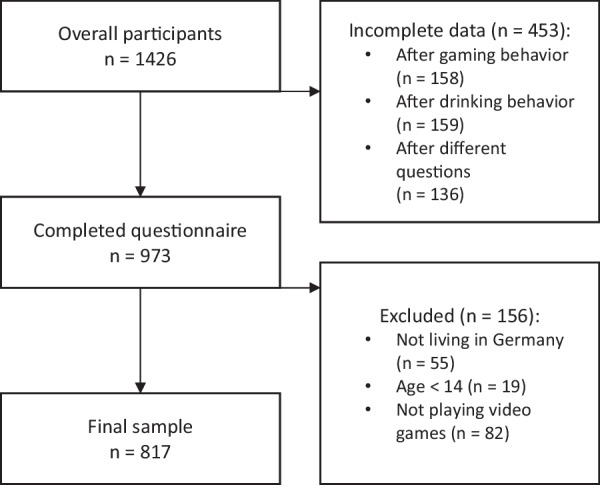
Flowchart to the analyzable data sets

In Fig. 2, the progress toward the final sample is displayed. Accordingly, the total sample consists of 817 participants, of whom 20 are professional esports players, 15 are former professional esports players, 190 are esports amateurs, 456 are regular players, and 136 are occasional players (Table 1). Hence, the participants can be divided into n = 607 video game players and n = 210 esports players.

**Table 1:** Sample characteristics

Group	Age^1^ [years] Mean (SD) Median (P25–P75)	Gender^2^ [“male”] n (%)	BMI^1^ [kg/m^2^] Mean (SD) Median (P25–P75)	Education^1^ [“higher education entrance qualification or higher”] n (%)	Employment^2^ [“full-time employment”] n (%)
Total sample (n = 808)	24.2 (6.9)	703 (87.0)	24.7 (5.1)	545 (67.5)	247 (30.6)
	23.0 (20.0–27.0)		24.0 (21.6–26.6)		
Professional players (n = 20)	22.8 (4.5)	19 (95.0)	24.0 (3.8)	15 (75.0)	7 (35.0)
	22.0 (20.0–23.8)		23.0 (21.0–26.2)		
Former professional players (n = 15)	28.4 (7.4)	14 (93.3)	25.3 (3.8)	13 (86.7)	10 (66.7)
	27.0 (22.0–37.0)		24.8 (22.3–26.6)		
Amateurs (n = 187)	24.0 (7.3)	178^R,O^ (95.2)	24.9 (4.9)	119 (63.6)	54 (28.9)
	22.0 (19.0–26.0)		24.2 (21.5–27.4)		
Regular players (n = 452)	23.9 (6.5)	392^A,O^ (86.7)	24.9 (5.5)	294 (65.0)	136 (30.1)
	23.0 (20.0–27.0)		24.2 (21.6–26.6)		
Occasional players (n = 134)	25.2 (7.6)	100^A,R^ (74.6)	24.0 (4.2)	104 (77.5)	40 (29.9)
	23.0 (21.0–27.0)		23.5 (21.4–25.8)		
**p**	0.06	** < 0.01**	0.32	**0.04**	0.06

^1^Kruskal–Wallis test; ^2^Fisher’s Exact Test; 9 participants are missing, because of not specifying gender, weight or height. Superscript letters indicate statistically significant (p < 0.05) differences to other groups in the same column: ^P^professional players; ^F^former professional players; ^A^amateurs; ^R^regular players; ^O^occasional players. Bold values shows a significance (p < 0.05)

Table 1 displays the characteristics of the sample regarding player status. On average, the total sample is mostly young (24.2 ± 6.9 years), predominantly male (87.1%), and well educated (67.5% have at least a higher education entrance qualification). The majority of the sample are students (39.3%) or work in full-time employment (30.6%). Pupils (11.9%) and trainees (8.4%) make up only a small proportion. The adults of the sample (19 years and older) have an average BMI of 25.1 ± 5.1 kg/m^2^ (n = 683), which can be classified as overweight. The percentage of female gamers differs between video game players (15.7%) and esports players (4.8%, p < 0.01). The Kruskal–Wallis test showed differences in education across groups (p = 0.04). However, according to the post hoc test with adjusted significance, no differences were found between the individual groups. There are no more significant differences between the video game players and esports players based on the sample characteristics.

### Health behavior

Table 2 displays the data on the health behavior of the sample. Overall, the participants reported their health status mostly as “excellent” (16.8%), “very good” (43.9%), or “good” (33.0%). Only the minority had a “fair” or “poor” (6.2%) health status. On average, the sample is physically active for 9.2 ± 8.4 h a week. In contrast, the participants are sedentary for 7.7 ± 3.6 h a day. For the WHO-5 questionnaire, the sample had an average score of 59.4 ± 17.5. When it comes to playing time, the professional players play the most (36.4 ± 23.7 h/week).

**Table 2:** Data on health behavior of the sample

Group	Health Status^1^ [Mode] n (%) Median (P25–P75)	Physical Activity^1^ [hours/week] Mean (SD) Median (P25–P75)	Sedentary time^1^ [hours/day] Mean (SD) Median (P25–P75)	WHO-5 Score^1^ [1–100] Mean (SD) Median (P25–P75)	Playing time [hours/week] Mean (SD) Median (P25–P75)
Total sample (n = 800)	“very good” 352 (43.9)	9.2 (8.4)	7.7 (3.6)	59.4 (17.5)	20.3 (15.6)^O^
	4.0 (3.0–4.0)	7.0 (4.0–10.0)	8.0 (5.0–10.0)	60.0 (48.0–72.0)	17.0 (0.9–27.0)
Professional players (n = 20)	“very good” 10 (50.0)	7.0 (5.6)	7.9 (4.1)	69.2 (9.6)	36.4 (23.7)
	3.0 (3.3–4.8)	5.5 (4.0–9.5)	8.0 (4.3–11.5)	68.0 (64.0–72.0)	33.5 (19.3–48.5)
Former professional players (n = 15)	“very good” & “good” 6 (40.0)	9.8 (6.3)	8.5 (3.5)	64.5 (8.0)	23.0 (16.7)^O^
	4.0 (3.0–4.0)	8.0 (5.0–12.0)	8.0 (5.0–12.0)	64.0 (56.0–72.0)	18.0 (11.0–40.0)
Amateurs (n = 187)	“very good” 80 (42.8)	8.4 (8.2)^O^	8.3 (3.6)^O^	60.8 (16.6)	26.7 (14.9)^O,R^
	4.0 (3.0–4.0)	6.0 (3.0–10.0)	9.0 (6.0–10.0)	64.0 (52.0–72.0)	23.0 (16.0–35.0)
Regular players (n = 445)	“very good” 197 (44.1)	9.3 (8.8)	7.8 (3.7)^O^	58.7 (17.8)	21.3 (14.3)^O,A^
	4.0 (3.0–4.0)	7.0 (4.0–10.0)	8.0 (5.0–10.0)	60.0 (48.0–72.0)	18.0 (11.0–26.5)
Occasional players (n = 133)	“very good” 59 (44.4)	10.2 (7.6)^A^	6.5 (2.8)^R,A^	57.8 (18.8)	5.1 (5.6)^P,F,A,R^
	4.0 (3.0–4.0)	8.0 (5.0–14.0)	6.0 (4.0–8.0)	60.0 (48.0–72.0)	3.5 (2.0–7.0)
**p**	0.55	**0.03**	** < 0.01**	0.05	** < 0.01**

^1^Kruskal–Wallis test; 17 Participants missing, because of not specifying physical activity, sedentary time. Superscript letters indicate statistically significant (p < 0.05) differences to other groups in the same column: ^P^professional players; ^F^former professional players; ^A^amateurs; ^R^regular players; ^O^occasional players. Bold values shows a significance (p < 0.05)

In comparison between the groups, esports players (8.25 ± 3.6 h/day) sit statistically significantly longer than video game players (7.5 ± 3.5 h/day, p < 0.01). Additionally, the esports players (8.3 ± 8.0 h/week) are significantly less physically active than the video game players (9.5 ± 8.5 h/week, p = 0.03). Regarding the playing time, all player categories playing statistically significant more than the occasional players (p < 0.01). Furthermore, the amateurs (26.7 ± 14.9 h/week) play more hours in a week than the regular players do (21.3 ± 14.3 h/week; p < 0.01).

### Drinking behavior

Table 3 displays the daily drinking consumption of the participants. The main source of fluid intake for them is water (10.9 ± 7.0 l/week). With the exception of beer (0.9 ± 1.6 l/week), the consumption of alcoholic beverages is rather low. Overall, nearly a quarter of the participants do not drink alcohol. Among those under 18 years old, half of them drink alcohol.

**Table 3:** Drinking consumption of the participants in servings a day

Drinking consumption [servings/day]	Total sample (n = 807) Mean (SD) Median (P25-P75)	Esports players (n = 207) Mean (SD) Median (P25-P75)	Video game players (n = 600) Mean (SD) Median (P25-P75)	p^1^
Water^a^	7.8 (5.0)	8.0 (5.3)	7.7 (4.8)	0.77
	8.0 (4.0–10.0)	8.0 (3.0–10.0)	7.0 (4.0–10.0)	
Juice^a^	0.7 (1.3)	0.8 (1.5)	0.7 (1.3)	0.83
	0.2 (< 0.1–0.6)	0.2 (0.0–0.8)	0.2 (< 0.1–0.6)	
Soft drinks^a^	0.9 (1.8)	1.0 (1.9)	0.8 (1.8)	0.73
	0.2 (< 0.1–0.9)	0.2 (< 0.1–1.0)	0.2 (< 0.1–0.7)	
Energy drinks^b^	0.2 (0.5)	0.2 (0.6)	0.1 (0.4)	**0.02**
	0.0 (0.0–0.1)	0.0 (0.0–0.2)	0.0 (0.0–0.1)	
Light-softdrinks^a^	0.4 (1.2)	0.4 (1.2)	0.4 (1.3)	0.49
	0.0 (0.0–0.2)	0.0 (0.0–0.2)	0.0 (0.0–0.2)	
Coffee^c^	0.9 (1.4)	0.9 (1.5)	0.9 (1.4)	0.74
	0.1 (0.0–1.6)	0.1 (0.0–1.6)	0.1 (0.0–1.6)	
Tea^c^	0.5 (1.2)	0.5 (1.1)	0.5 (1.2)	0.93
	< 0.1 (0.0–0.4)	< 0.01 (0.0–0.4)	< 0.1 (0.0–0.4)	
Beer^e^	0.4 (0.7)	0.4 (0.8)	0.5 (0.7)	0.11
	0.2 (0.0–0.5)	0.1 (0.0–0.4)	0.2 (0.0–0.6)	
Wine^d^	0.1 (0.2)	< 0.1 (0.1)	0.1 (0.2)	0.08
	0.0 (0.0– < 0.1)	0.0 (0.0–0.0)	0.0 (0.0– < 0.1)	
Mixed alcoholic drinks^a^	0.1 (0.3)	0.1 (0.3)	0.1 (0.3)	0.53
	0.0 (0.0–0.1)	0.0 (0.0–0.1)	0.0 (0.0–0.1)	
Spirits^f^	0.1 (0.3)	0.1 (0.3)	0.1 (0.2)	0.71
	0.0 (0.0–0.1)	0.0 (0.0–0.1)	0.0 (0.0–0.1)	
Other alcoholic drinks^a^	< 0.1 (0.2)	< 0.1 (0.2)	< 0.1 (0.2)	0.13
	0.0 (0.0–0.0)	0.0 (0.0–0.0)	0.0 (0.0–0.0)	
Other alcoholic free drinks^a^	0.4 (1.7)	0.6 (2.2)	0.4 (1.6)	0.16
	0.0 (0.0– < 0.1)	0.0 (0.0–0.0)	0.0 (0.0–0.1)	

^1^Mann–Whitney U test; 10 Participants missing, because of not specifying at least one information for the drinks; ^a^serving = 200 ml, ^b^serving = 250 ml, ^c^serving = 150 ml, ^d^serving = 125 ml, ^e^serving = 330 ml, ^f^serving = 20 ml. Bold values shows a significance (p < 0.05)

Except for energy drinks, no statistically significant differences in drinking consumption were found between video game players and esports players. Esports players (0.2 ± 0.6 servings/day) drink significantly more energy drinks than the video game players (0.1 ± 0.4 servings/day; p = 0.02).

### Dietary behavior

Most of the participants (85.2%) do not follow a specific dietary pattern. The other participants (14.8%) are vegetarians or vegans. Overall, nearly half of the gamers prepare their own meals at least five times a week. No statistically significant differences were found between the video game players and esports players.

Table 4 displays the dietary behavior of the participants. While bread or buns are eaten every day (1.6 ± 1.2 servings/day), other carbohydrate foods are eaten only several times a week. There are no statistically significant differences between the groups.

**Table 4:** Dietary behavior of the participants in servings a day

Dietary behavior[servings/day]	Total sample (n = 807) Mean (SD) Median (P25–P75)	Esports players (n = 210) Mean (SD) Median (P25–P75)	Video game players (n = 597) Mean (SD) Median (P25–P75)	p^1^
Bread/Buns^a^	1.6 (1.2)	1.6 (1.1)	1.6 (1.3)	0.83
	1.5 (0.5–2.0)	1.6 (0.6–2.0)	1.5 (0.5–2.0)	
Pasta^b^	0.5 (0.6)	0.6 (0.6)	0.5 (0.6)	0.37
	0.4 (0.2–0.6)	0.4 (0.2–0.7)	0.4 (0.2–0.6)	
Rice^c^	0.4 (0.5)	0.5 (0.6)	0.4 (0.5)	0.15
	0.2 (0.1–0.4)	0.2 (0.1–0.4)	0.2 (0.1–0.4)	
Cereals^d^	0.3 (0.6)	0.3 (0.6)	0.3 (0.6)	0.06
	< 0.1 (0.0–0.4)	0.0 (0.0–0.3)	0.1 (0.0–0.4)	
Cornflakes^d^	0.0 (0.4)	0.2 (0.5)	0.1 (0.4)	1.0
	0.0 (0.0–0.1)	0.0 (0.0–0.1)	0.0 (0.0–0.1)	
Fruits^e^	0.9 (1.0)	0.9 (1.0)	1.0 (1.1)	0.10
	5.0 (0.2–1.1)	0.5 (0.2–1.0)	0.5 (0.2–1.5)	
Vegetables^e^	1.7 (1.6)	1.6 (1.3)	1.7 (1.7)	0.52
	1.5 (0.5–2.4)	1.5 (0.5–2.1)	1.5 (0.5–2.4)	
Potatoes^e^	0.6 (0.8)	0.6 (0.9)	0.6 (0.7)	0.42
	0.4 (0.2–0.6)	0.4 (0.2–0.9)	0.4 (0.2–0.6)	
Nuts^f^	0.2 (0.4)	0.2 (0.4)	0.2 (0.4)	0.64
	0.1 (0.0–0.3)	0.1 (0.0–0.2)	0.1 (0.0–0.3)	
Red meat^g^	0.5 (0.6)	0.6 (0.7)	0.5 (0.5)	**0.02**
	0.2 (0.1–0.6)	0.4 (0.2–0.8)	0.2 (0.1–0.5)	
Poultry^g^	0.4 (0.5)	0.5 (0.6)	0.3 (0.5)	** < 0.01**
	0.2 (0.1–0.4)	0.2 (0.1–0.5)	0.2 (0.1–0.4)	
Fish^g^	0.1 (0.2)	0.2 (0.3)	0.1 (0.2)	0.08
	0.1 (0.0–0.2)	0.1 (0.0–0.2)	0.1 (0.1–0.8)	
Sausage^h^	1.0 (1.4)	1.1 (1.5)	1.0 (1.4)	0.35
	0.4 (0.0–1.5)	0.4 (0.0–1.6)	0.4 (0.0–1.5)	
Cheese^h^	0.8 (1.0)	0.7 (1.2)	0.8 (1.0)	**0.03**
	0.4 (< 0.1–1.0)	0.4 (0.0–1.0)	0.4 (0.1–1.0)	
Dairy produtcs^j^	0.5 (0.8)	0.4 (0.6)	0.6 (0.9)	** < 0.01**
	0.2 (< 0.1–0.6)	0.2 (0.0–0.5)	0.2 (0.1–0.8)	
Fast food^k^	0.3 (0.4)	0.3 (0.4)	0.2 (0.4)	** < 0.01**
	0.2 (0.1–0.3)	0.2 (0.1–0.4)	0.2 (0.1–0.3)	
Sweet bakery products^l^	0.2 (0.3)	0.2 (0.3)	0.2 (0.3)	** < 0.01**
	0.1 (0.0–0.2)	0.1 (0.0–0.2)	0.1 (< 0.1–0.2)	
Chips & savory snacks^m^	0.2 (0.4)	0.2 (0.5)	0.2 (0.3)	0.10
	0.1 (0.0–0.2)	0.1 (0.0–0.2)	0.1 (0.0–0.2)	
Sweets^n^	0.6 (0.8)	0.5 (0.8)	0.6 (0.9)	** < 0.01**
	0.3 (0.1–0.9)	0.2 (< 0.1–0.5)	0.3 (0.1–1.0)	

^1^Mann–Whitney U test; 10 participants missing, because of not specifying at least one information of the foods; ^a^serving = slice/bun, ^b^serving = one plate (100 g raw/200 g cooked), ^c^serving = small cup (50 g raw), ^d^serving = small bowl (150 g), ^e^serving = fist sized piece, ^f^serving = handful, ^g^serving = filet, ^h^serving = slice, ^j^serving = cup (150 g), ^k^serving = dish, ^l^serving = piece, ^m^serving = small bowl (125 g), ^n^serving = candy bar (25 g). Bold values shows a significance (p < 0.05)

The results show that both vegetables (1.7 ± 1.6 servings/day) and fruits (0.9 ± 1.0 servings/day) are consumed almost daily. For these foods, there are no significant differences between the groups.

To have a better idea of the serving amounts, animal-based products are given in servings per month here. It shows that the esports players (16.4 ± 18.5 servings/month) eat more red meat than the video game players (13.4 ± 15.6 servings/month; p = 0.02). Additionally, esports players consume 12.8 ± 16.6 servings of poultry a month, while video game players eat 9.7 ± 13.7 servings a month (p < 0.01). In contrast, video game players consume statistically significantly more dairy products and cheese than esports players.

The esports players (9.1 ± 11.6 servings/month) consume statistically significantly more fast food than the video game players (7.0 ± 10.0 servings/month; p < 0.01). However, the video game players (18.2 ± 26.5 servings/month) eat statistically significantly more sweets than the esports players (14.1 ± 22.0 servings/month; p < 0.01). The same applies to sweet bakery products.

### Body image

Table 5 displays the desired and current body image of the participants. Due to low number of the participants with diverse genders (n = 9), they were not considered in the following section. On average, women indicated a 3.0 ± 0.9 (n = 105) for their desired body image (see Fig. 1) and a 4.0 ± 1.4 for their current body image. On the other hand, the male participants desired a body image of 3.9 ± 0.9 (n = 695) and had a current body image of 4.3 ± 1.5. Accordingly, the body image of the female participants deviates on average by 1.0 ± 1.1 points and for the men by 0.5 ± 1.2 points, whereby in each case the leaner body image was desired. Accordingly, there are significant differences between the gender in the deviations of the desired body image and the current body image (p < 0.01). But there are also significant differences between the current body image and desired body image within both male and female participants (p < 0.01).

**Table 5:** The desired and current body image of the participants

	Male		Female	
	Desired body image Mean (SD) Median (P25–P75)	Current body image Mean (SD) Median (P25–P75)	Desired body image Mean (SD) Median (P25–P75)	Current body image Mean (SD) Median (P25–P75)
Total sample (Male: n = 695) (Female: n = 105)	3.9 (0.9) 4.0 (3.0–4.0)	4.3 (1.5) 4.0 (3.0–5.0)	3.0 (0.9) 3.0 (2.0–4.0)	4.0 (1.4) 4.0 (3.0–5.0)
Video game players (Male: n = 502) (Female: n = 95)	3.8 (0.9) 4.0 (3.0–4.0)	4.3 (1.5) 4.0 (3.0–5.0)	3.0 (1.0) 3.0 (2.0–3.0)	4.0 (1.5) 4.0 (3.0–5.0)
Esports players (Male: n = 193) (Female: n = 10)	4.0 (0.8) 4.0 (4.0–5.0)	4.5 (1.6) 4.0 (3.5–5.5)	3.3 (0.7) 3.0 (3.0–4.0)	3.8 (0.8) 4.0 (3.8–4.0)
p	** < 0.01**		** < 0.01**	

Sign test. n = 800 due to missing data. Bold values shows a significance (p < 0.05)

There are no significant differences between video game players and esports players (p = 0.56). Even if the groups are divided by gender, there are no significant differences between them (male: p = 0.64; female: p = 0.09).

### Associations with health status and video game playing time

Table 6 displays significant partial spearman correlations with health status as well as video game playing time and health behaviors, drinks, and nutrition. Several poor to moderate correlations were found. Although parameters such as video game playing time and beverages, with the exception of energy drinks, show significant associations, the correlations are too weak to be relevant. Foods and beverages not included in the table have no significant correlation with the health status.

**Table 6:** Correlations with health status and video game playing time

	Health status		Video game playing time	
	rho	p	rho	p
Well-being score	**0.42**	** < 0.01**	− 0.02	0.61
Physical activity	**0.31**	** < 0.01**	− 0.02	0.63
Sedentary time	**0.21**	** < 0.01;**	**0.23**	** < 0.01**
Health status			− 0.09	0.01
BMI	− **0.31**	** < 0.01**	0.05	0.13
Video game playing time	− 0.08	0.02		
Water	0.08	0.03	− 0.07	0.04
Soft drinks	− 0.08	0.03	**0.15**	** < 0.01**
Energy drinks	− **0.11**	** < 0.01**	**0.14**	** < 0.01**
Light soft drinks	− 0.09	0.01	− 0.03	0.45
Coffee	0.62	0.02	− **0.13**	** < 0.01**
Beer	0.04	0.23	− **0.12**	** < 0.01**
Wine	0.09	0.01	− **0.16**	** < 0.01**
Mixed alcoholic drinks	0.08	0.04	− **0.10**	** < 0.01**
Fruits	**0.19**	** < 0.01**	− **0.14**	** < 0.01**
Vegetables	**0.14**	** < 0.01**	− **0.10**	** < 0.01**
Cereals	**0.12**	** < 0.01**	− **0.15**	** < 0.01**
Cornflakes	0.05	0.17	**0.12**	** < 0.01**
Fish	**0.11**	** < 0.01**	− 0.01	0.71
Dairy products	0.09	< 0.01	− **0.14**	** < 0.01**
Cheese	− 0.04	0.28	− 0.09	0.02
Nuts	**0.10**	** < 0.01**	− **0.10**	** < 0.01**
Fast food	− **0.12**	** < 0.01**	**0.13**	** < 0.01**
Red meat	0.01	0.88	0.09	< 0.01
Poultry	− 0.01	0.83	**0.11**	** < 0.01**
Sausage	− 0.08	0.03	0.08	0.02
Sweet bakery products	0.04	0.25	− **0.12**	** < 0.01**

Partial spearman correlations. n = 789 due to missing data in the included parameters. Bold lines show at least a significant weak correlation

The findings show a positive but weak relationship between playing time and sedentary time per day (rho = 0.23; p < 0.01). There are also poor positive correlations between playing time and energy drinks (rho = 0.14; p < 0.01) as well as fast food (rho = 0.13; p < 0.01). Apart from the correlations shown in the table, there are no other statistically significant correlations.

### Correlations with the body image

Table 7 shows correlations between the differences in body image and parameters of the health behaviors. There is a moderately negative correlation between the difference of the desired and current body image and health status (p < 0.01; rho = −0.35). Correlations between the difference in participants' current and desired body image and their dietary behavior were not calculated due to a lack of significance.

**Table 7:** Correlations with body image

	Body image
Well-being score	**p < 0.01; rho = −0.24**
Physical activity	**p < 0.01; rho = −0.13**
Sedentary time	**p < 0.01; rho = 0.16**
BMI	**p < 0.01; rho = 0.33**
Video game playing time	**p < 0.01; rho = 0.11**
Health status	**p < 0.01; rho = −0.35**

Partial spearman correlations. n = 794 due to missing data in the included parameters. Bold lines show at least a significant weak correlation

## Discussion

### Key results

The aim of the eSports Study 2021 was to investigate the health and dietary behavior of video game players and esports players in Germany. Data from over 800 mostly male, young, and well-educated participants show that self-reported health is generally good. While the players have relatively high sitting times, the majority are also physically active. Nevertheless, adults are slightly overweight on average. Dietary behavior shows no major abnormalities compared to the general German population, except for the energy drinks. The high consumption poses a health risk due to the high sugar content, among other things. Overall, the participants are mostly satisfied with their body image.

### Demography and health behavior

As shown in previous studies, gamers in Germany are predominantly male, young, and well-educated [21, 22]. The vast majority of participants are students or have full-time jobs. Besides, they pursue their hobby of gaming, which, due to its nature, is played mostly while they sit. In comparison to other studies, the participants in this study played nearly the same amount of video games, approximately 20 h per week [4]. The high playing times of the professionals (36 h per week) are probably related to the fact that they earn their living with the video games and therefore have to invest more time in them. Due to the overall high playing times, the players have high sitting times, which are, however, comparable to the German population [29, 30]. Nevertheless, even though there is currently no evidence to recommend exact sitting times, these should be reduced [31]. The body mass index of the adult participants can be classified as overweight, but it is also comparable to the German population, which has an BMI of 26.0 kg/m^2^ [32]. In this respect, studies have already shown that gaming is not related to an increased BMI [33]. As in previous studies, the majority of gamers (85.3%) exceed the WHO’s recommendations of 2.5 h a week of moderate to vigorous physical activity. In Germany an average of 42.6% of female and 48.0% of male people reach the WHO recommendations. [34]. Therefore, a larger proportion reaches the recommendations than the German population. However, the sample of the present study is younger than the normal population, and physical activity often decreases with age [35]. Moreover, these are only the minimum recommendations [36]. For additional health benefits, more than 300 min of moderate physical activity and two muscle-strengthening activities per week are necessary. The results cannot provide any information on this because the type of physical activity was not surveyed. According to the data, the stereotype of the sedentary and physically inactive gamer cannot be supported.

Esports players reported a quiet, positive general health status. But there are significant differences between the desired and actual body image. However, on average this only differs in a maximum of one point, so for the most part they seem to be satisfied with their body image. Being satisfied with the own body is very important for well-being and overall health [37]. Regarding the WHO-5, the majority also show good psychological well-being. On average, the scores of the participants were above 50, which is considered the cut-off score, for precarious well-being [38]. Compared to amateur and professional athletes in traditional sports, the esports players showed similar scores [39]. However, it is a serious matter that almost a quarter of the participants were below the cut-off score and thus further psychological diagnostic procedures should be performed with regard to depression. Compared to previous studies, the well-being of video game and esports players decreased [21, 22]. One reason for the decreasing well-being could be the COVID-19 pandemic that occurred at the time of data collection. As some studies have shown, there has been a general decline in mental health and well-being in the population [40, 41]. Accordingly, future research should look more closely at the well-being of video game players and esports players.

### Drinking behavior

With 1.6 L per day, water is the main source of fluid for the participants. This is above the general recommendations of the German Nutrition Society (DGE), which recommends 1.5 L per day [42]. The majority (91.3%) achieves the recommendation of 1.5 L a day. This puts them on par with 11–17 year-olds living in Germany, who drink 1.4 L per day [43]. However, the individual water intake depends on factors such as physical composition and activity during the day. Accordingly, 1.5 L is the minimum recommendation. Depending on age, weight, and physical activity, more water should be drunk [44].

Although it is recommended that water should be used to meet the liquid requirement, other beverages can also be consumed. Very popular in this context are soft drinks. Compared to the 14–17 year olds, who drink an average of almost half a liter of soft drinks per day, the participants in this study drink clearly less [45]. If adding the juice consumption, which contain nearly as much sugar as the soft drinks, the participants consume only half as many soft drinks and juice as the German 11–17 year olds, who drink about 573 ml per day [43]. Older age groups also consume more of these beverages. For example, men aged 18–29 drink an average of 4.5 glasses of juice and soft drinks per day [46]. Nevertheless, the consumption of gamers at one serving per day is already too high and should be decreased. The high sugar content of these drinks can lead to an increased risk of diabetes, weight gain, and high blood pressure [19, 47].

Similar reasoning applies to energy drinks. On average, participants drink more than one can per week. Compared to the German population, in which only just under 10% drink a can at least once a week, the players in this study drink much more energy drinks [48]. In comparison to the other groups, esports players drink significantly more. This could be related to the prevalent sponsorships in esports. Energy drinks are also advertised in a way to provide health benefits [49], especially for increasing performance and concentration, which are particularly relevant in esports. However, besides the high amount of sugar, energy drinks have further health risks. According to this, insomnia, stress, depressive mood, and gastrointestinal upset could be observed with regular consumption [50]. Nevertheless, an overall reduction of sugary beverages is to be strived for, because these are associated with obesity and diseases such as diabetes [14, 15, 51]. The association between energy drinks and health status in this study strengthens the explanation.

Because the number of studies that investigated the consumption of energy drinks in traditional sports is limited, it is difficult to draw a comparison with them. Nonetheless, one study [52] looked at energy drink consumption in young athletes as young as 14 years old. Seventeen percent of them drank energy drinks daily or one to two times a week. While the authors could not show a correlation with time spent exercising, another study showed that people who exercise more in their leisure time also consume more energy drinks [53]. However, the values are also hardly comparable with the present study.

In contrast, the consumption of alcoholic beverages among video game players and esports players is low. About a quarter of the participants do not drink alcohol at all. Compared to the per capita consumption per year of the German population, gamers drink less. Beer alone is already drunk by the German population about 0.2 l per day [54]. In context, according to the German Nutrition Society, the tolerable amount of alcohol for healthy men is 20 g per day [42]. This corresponds to about half a liter of beer or 250 ml of wine. The average consumption of both the total sample and the groups of video game players and esports players are below this limit.

### Dietary behavior

In terms of diet, gamers are very similar to the German population, with one-tenth following a vegetarian or vegan diet [55]. In contrast, the participants, with more than six servings a week, eat relatively much meat. In addition, they eat a slice of sausage on average every day. The German Nutrition Society recommends only 300–600 g of meat per week, which is equivalent to two to four servings [42]. Eating too much meat and sausage is associated with a higher risk of colorectal cancer [42, 56]. Fish should be eaten once a week because of its omega-3-fatty acids [57]. With just under one meal per week, the participants achieve this recommendation.

Dairy products are necessary to ensure adequate calcium intake. Gamers, on the other hand, consume less than the recommended amount of 200–250 g milk and two slices of cheese. Overall, the diet is largely carbohydrate-heavy. Several slices of bread, rice, pasta and potatoes are consumed almost daily. Due to the positive effects on the gut, it is recommended to consume whole grain products made from it [58]. In addition, whole grain products are associated with a lower incidence of cardiovascular disease [18], cancer [59], and type 2 diabetes [60]. However, this study did not ask about the quality of carbohydrates. Accordingly, no statement can be made about the type of consumed carbohydrates. For a balanced diet, vegetables and fruits are also necessary. They are an important source of vitamins, minerals, trace elements, and fiber [61]. The German Nutrition Society recommends “five a day,” which means that at least three servings of vegetables and two servings of fruits should be eaten every day [42, 62]. One serving of nuts or one glass of juice can replace one serving of fruit [62]. According to the results, only a small percentage of 16% achieves these recommendations. The video game players reach them significantly more often than the esports players do. Compared to the German population gamers perform minimally better. In Germany, only 15% of women and 7% of men reach recommendations for fruits and vegetables [61]. Nevertheless, there is an overall lack of vegetable and fruit consumption. Especially with regard to the prevention of chronic diseases such as stroke, heart disease, and hypertension, attention should be paid to increasing vegetable and fruit consumption [63, 64].

Even if the consumption of sweets and chips is quite low, the participants like to resort to quick meals such as fast food. Almost two meals per week consist of it. Due to the negative association with subjective health in this study, fast food consumption should be limited. Even though it is best to consume less, the amount compared to 12–17 year old Germans is similar. They consume about 400–600 g of fast food per week [65]. These results are also reflected in the cooking frequency of the participants. Nearly half of the participants cook for themselves five times a week. This is close to the 14–29 year old German population, in which half cook almost every day [55]. However, considering that participants were forced to cook for themselves during the COVID-19 pandemic due to lockdowns, this is only a small proportion of those who actually cook regularly.

### Limitations

This study also has some limitations. First, because the current study is based solely on self-reported data, the results may be influenced by social desirability or subjective over- and underestimation of certain behaviors such as sedentary times, physical activity, or food and drink consumption [66] Especially when specifying the amount of nutrition, it might be difficult to remember exactly or to estimate the servings correctly. To minimize these effects, the data were checked for paradoxical entries or extreme values. However, the effects of social desirability and inaccurate reporting cannot be ruled out in general.

Secondly, the COVID-19 pandemic and the accompanying security measures had a significant impact on the population and therefore possibly also on dietary behavior. On the one hand, restaurants were closed during the lockdown, which meant that people were increasingly forced to cook for themselves. In addition, sports in clubs or studios were limited, which affected the sports opportunities and therefore possibly the physical activity. On the other hand, digital media got a boost, which increased screen time and, in particular, video game playing time.

Thirdly, questions about the characteristics of video gamers (like playing time, sitting time, physical activity, etc.) have been largely self-generated. Either there are no validated questionnaires on this yet or the processing time should be kept as short as possible to reduce the dropout rate. This could have led to inaccurate data. For example, the question on physical activity is based on a simple indication in minutes and hours. For more valid results in future, questionnaires like the IPAQ short form or EHIS-PAQ should be used. Overestimation, misreporting, or the effect of social desirability cannot be ruled out. Accordingly, the high proportion of physical activity compared to other population groups is questionable. Insofar as length and processing time allow, validated questionnaires should be used. The food frequency questionnaire was also slightly adapted, and some foods were combined into one. While portion sizes were included with all foods, everyone could subjectively perceive them differently. However, the results still provide a good overview of the approximate dietary behavior of video game players and esports players.

## Conclusions

Overall, video game players and esports players reported good general health and well-being, as well as some healthy habits. The gamers seem to eat very similarly to the German population. Nevertheless, there is a need for overall improvement overall. Energy drinks, in particular, can have a negative impact on health and lead to obesity or diseases such as diabetes in the long run. Among other things, sponsors in the industry are responsible for the high consumption. These sponsorships should be critically scrutinized for the possible health consequences of their products. In addition to limiting energy drinks and fast food, the positive effects of fruits and vegetables on health and performance should be highlighted. To keep the target group healthy, they should receive sufficient support and education in nutrition as early as possible.

## Data Availability

The datasets used and/or analyzed during the current study are available from the corresponding author on reasonable request.

## Abbreviations

BMI: Body mass index
e.g.: For example
WHO: World Health Organization
